# Shortcomings in the Evaluation of Blood Glucose Forecasting

**DOI:** 10.1109/TBME.2024.3424665

**Published:** 2024-11-21

**Authors:** Jung Min Lee, Rodica Pop-Busui, Joyce M. Lee, Jesper Fleischer, Jenna Wiens

**Affiliations:** Division of Computer Science and Engineering, University of Michigan, USA.; Department of Internal Medicine, Division of Metabolism, Endocrinology and Diabetes, University of Michigan, USA. She is now with the Division of Endocrinology, Diabetes and Clinical Nutrition, Harold Schnitzer Diabetes Center, Oregon Health Science University, USA.; Susan B. Meister Child Health Evaluation and Research Center, Division of Pediatric Endocrinology, University of Michigan, USA.; Steno Diabetes Center Aarhus, Denmark, and also with the Steno Diabetes Center Zealand, Denmark.; Division of Computer Science and Engineering, University of Michigan, 48109 USA

**Keywords:** Artificial intelligence, artificial pancreas, blood glucose forecasting, closed-loop systems, forecasting evaluation, machine learning

## Abstract

**Objective:**

Recent years have seen an increase in machine learning (ML)-based blood glucose (BG) forecasting models, with a growing emphasis on potential application to hybrid or closed-loop predictive glucose controllers. However, current approaches focus on evaluating the accuracy of these models using benchmark data generated under the behavior policy, which may differ significantly from the data the model may encounter in a control setting. This study challenges the efficacy of such evaluation approaches, demonstrating that they can fail to accurately capture an ML-based model’s true performance in closed-loop control settings.

**Methods:**

Forecast error measured using current evaluation approaches was compared to the control performance of two forecasters – a ML-based model (LSTM) and a rule-based model (Loop) – *in silico* when the forecasters were utilized with a model-based controller in a hybrid closed-loop setting.

**Results:**

Under current evaluation standards, LSTM achieves a significantly lower (better) forecast error than Loop with a root mean squared error (RMSE) of 11.57 ± 0.05 mg/dL vs. 18.46 ± 0.07 mg/dL at the 30-minute prediction horizon. Yet in a control setting, LSTM led to significantly worse control performance with only 77.14% (IQR 66.57–84.03) time-in-range compared to 86.20% (IQR 78.28–91.21) for Loop.

**Conclusion:**

Prevailing evaluation methods can fail to accurately capture the forecaster’s performance when utilized in closed-loop settings.

**Significance:**

Our findings underscore the limitations of current evaluation standards and the need for alternative evaluation metrics and training strategies when developing BG forecasters for closed-loop control systems.

## I. Introduction

TYPE 1 diabetes (T1D) is a chronic autoimmune disease characterized by the progressive damage and loss of pancreatic beta-cells leading to a lack of endogenous insulin [1]. Due to the inability to produce insulin, people with T1D require constant exogenous insulin administration to maintain their blood glucose (BG) levels within a normal range (70–180 mg/dL). This can be a significant cognitive burden, as it requires frequent monitoring of BG levels and calculation of insulin boluses at every meal [2]. Developments such as continuous glucose monitoring (CGM) technology and insulin pumps have helped alleviate some of this burden by providing real-time monitoring of BG levels and automating the delivery of pre-programmed insulin doses. The ultimate goal is to develop a fully automated system known as the artificial pancreas (AP), thereby entirely relieving the cognitive burden of users [3]. Although a fully closed-loop system is yet to be realized, researchers have made progress with systems that support and partially automate the decision-making process. One example is the bolus advisor, an algorithm designed to suggest optimal bolus insulin doses at mealtimes [4].

Model-based control (MBC) algorithms are a popular type of control algorithm that has been explored for this purpose [3]. As the name suggests, MBC algorithms use a forecasting model to predict the effects of candidate actions (e.g., different doses of insulin) on blood glucose levels. The algorithm then selects the best action based on these predictions while optimizing for some criteria (e.g., maximizing time in range), a process known as planning. To select the correct action, the model must accurately forecast the effect of candidate actions. However, creating an accurate BG forecasting model is challenging due to the non-linear dynamics of the glucoregulatory system, as well as the inter- and intra-patient variance in physiology [5], [6], [7]. In recent years, machine learning (ML) models have been increasingly applied to this task given their ability to model non-linear dynamics and adapt to changing domains [8], [9], [10], [11].

Despite the widespread adoption of ML-based glucose forecasters, evaluation of these models has focused on settings where the datasets used for training and evaluation were collected under a nearly deterministic behavior policy [12], [13], [14]. Specifically, BG forecasters are widely trained and evaluated on datasets gathered in open-loop settings. In contrast to closed-loop settings where insulin delivery is entirely automated, open-loop settings require users to determine the timing and dose of insulin delivery by relying on dosing regimens such as the basal-bolus policy. Here, the relationship between the amount of carbohydrates consumed and insulin administered is highly correlated and often nearly linear due to the simple, deterministic formula users rely on to calculate the size of prandial insulin doses (i.e., bolus insulin). Such data do not necessarily reflect the data the forecaster may encounter when deployed for control purposes. In control settings, the ability to generate accurate predictions for all candidate actions (e.g., cases where the carbohydrate and insulin amounts are not correlated) is required.

We hypothesize that this shortcoming of current evaluation approaches can lead to the selection of ML forecasters that seem accurate, and yet lead to poor control performance when utilized for control purposes. Unlike physiological models that explicitly encode the effect of each variable into the model [15], ML models focus on identifying patterns in the dataset and can fail when forced to make predictions on data outside of what they have seen during training. While the problem of poor generalization outside of the training distribution and its effect on planning has been studied in other domains [16], [17], addressing this problem within BG forecasting has been limited. For example, Finan et al. first highlighted this problem with linear dynamic models [18], and subsequent efforts have been made to mitigate this issue through transforming the inputs [19] or by creating physiologically-grounded models [20]. Others have also stressed the importance of using explainability tools to identify forecasters that may have erroneously learned a spurious relationship between carbohydrates and insulin [21], [22].

Despite these efforts, the adverse impact of the high collinearity between insulin and carbohydrates on ML forecasters is vastly underestimated among researchers in the ML community who aim to apply state-of-the-art ML techniques to problems in BG management. ML researchers who are unfamiliar with this issue continue to rely on flawed measures of accuracy in evaluating BG forecasters. This is evidenced by recent papers that have relied on such evaluation methods to select BG forecasters for the purpose of model-based control [7], [23].

In contrast to prior work, we focus specifically on the shortcomings of the current evaluation methods used to validate BG models. We probe the limitations of commonly used accuracy metrics by comparing the forecast error of the models under the behavior policy vs. during planning, and examine the relationship between forecast error and downstream control performance of models. We utilized an FDA-approved simulator and state-of-the-art BG forecasting models for our experiments. Our analysis highlights key shortcomings in the current approach to evaluating BG forecasters and the need for a more rigorous evaluation framework when selecting ML forecasters for model-based control.

## II. Methods

### A. Virtual Study Cohort

#### 1) Simulator:

Our experiments were conducted *in silico* using an open-source version of the UVA/Padova T1D simulator. The UVA/Padova simulator is based on a physiological model of the glucoregulatory system and includes a large virtual patient “cohort” that spans the variability of key parameters in the general population [24]. This cohort consists of participants in three groups (adults, adolescents, and children) each with patient-specific parameters designed to align with those observed in real patient data. The simulator has been approved by the FDA as a replacement for pre-clinical trials in evaluating closed-loop algorithms and has been used by dozens of research groups in academia [25]. In our experiments, we utilized the open-source version of the simulator [26] based on Python, as the proprietary version limits algorithms to be designed within a Matlab Simulink block. We selected 10 virtual adults from the cohort and paired the simulator with a realistic meal schedule (Appendix A). The simulator is equipped with a CGM, which measures the glucose level in the interstitial fluid. While this is a noisy proxy for BG, for simplicity we refer to this as BG throughout.

#### 2) Behavior Policy:

The standard dataset used for training and evaluating forecasters is generated using a behavior policy called the basal-bolus policy [13], [14]. This policy represents the self-management behaviors of individuals on insulin therapy. Under this policy, a low constant rate of basal insulin is delivered in the background while larger insulin boluses are administered to compensate for meals. The bolus size is calculated as:

$$bolus=\frac{CHO}{CR}+{𝟙}_{\left(b_{g}>150\right)}\frac{b_{g}-b_{t}}{ISF}$$

where CHO is the carbohydrate amount in grams, CR is the carbohydrate ratio, ISF is the insulin sensitivity factor, $b_{g}$ is the current BG, $b_{t}$ is the target BG (140 mg/dL) and $1_{\left(b_{g}>150\right)}$ is an indicator function that evaluates to 1 if the current BG is greater than 150 mg/dL and 0 otherwise. CR, ISF, and default basal rates were provided by the simulator. To mimic errors in carbohydrate estimation, CHO was set to between 80–120% of its true value, selected randomly from a uniform distribution. Boluses were set to occur anywhere between 15 minutes before and after a meal, based on a uniform distribution. We generated 100 days of data (10 rollouts of 10 consecutive days) for each individual under the behavior policy. This was split into 70, 15, and 15 consecutive days (per person) for training, validating, and testing the BG forecasters.

### B. Forecasting Models

We investigated two forecasting models: an ML-based model (LSTM) and a rule-based model (Loop). We hypothesized that the ML-based model would struggle in the MBC setting given the mismatch between the data encountered during training (behavior policy) and those encountered during control (MBC setting). For comparison, we considered a rule-based model that does not rely on a specific training set and incorporates domain knowledge regarding the causal effects of insulin and carbohydrates. We hypothesized that the rule-based model would be more robust to the data shift encountered in the MBC setting.

#### 1) LSTM:

Models based on LSTM networks have been utilized in many BG forecasting models [9], [27], [28], [29], and thus are our focus for ML-based models. We performed a thorough hyperparameter search to train LSTM models that generate a 4-hour long forecast. Hyperparameters included the amount of previous data used as input, the inclusion of insulin-on-board (IOB) and carbs-on-board (COB) estimates in the input, and the size of hidden states. As is common in the BG forecasting literature, we trained patient-specific models to minimize the root mean squared error (RMSE) between the predicted and true BG values within the forecast (details in Appendix B) [30]. Models achieving the best RMSE at the 4-hour prediction horizon on the validation set were selected and applied to the held-out test sets.

#### 2) Loop:

We used the forecaster provided in Loop, an open-source do-it-yourself AP system [31]. Loop generates a 6-hour long forecast that relies on several user-set parameters. We truncated this forecast to match the prediction length of LSTM when comparing the two models (i.e., used only the first 4 hours of the forecast). Total duration and peak time of insulin activity were set to 360 and 75 minutes respectively, the default setting recommended for adults. Meal absorption time was set to 1.5 hours. Retrospective correction and momentum effects were set as described in [31]. Note that, by design, Loop separately models the effects of carbohydrates and insulin.

### C. Model-Based Control (MBC) Algorithm

In model-based control a forecasting model is paired with a planning algorithm. We used random shooting (Algorithm 1) as the planning algorithm since it has been applied in a wide variety of continuous action tasks [32], [33], [34]. Given the bolus advisor setting, action selection was restricted to when a meal occurred. After each meal, 50 insulin boluses were randomly sampled from a uniform distribution between 0 and *maxbolus*. *maxbolus* is a patient-specific parameter and corresponds to the bolus the patient would receive if a meal contained 40% of their daily carbohydrate intake. For each bolus, the forecaster generated a 4-hour prediction corresponding to the expected BG trajectory given that dose along with the same default basal rate used by the behavior policy. The basal rate was kept consistent throughout the experiment. The bolus associated with the lowest predicted risk was selected. In assessing risk, we used the Magni risk function used in previous work [35], [36], which maps a single BG value b (in mg/dL) to a risk score:

$$MR\left(b\right)=10\times {\left(c_{0}\times {\left(\log\;b\right)}^{c_{1}}-c_{2}\right)}^{2}$$

where $c_{0}=3.35506$, $c_{1}=0.8353$, and $c_{1}=3.7932$. The overall risk score for trajectory $b_{t}=\left[b_{t},\;b_{t+1},\;\ldots ,\;b_{t+h-1}\right]$ is then defined as the cumulative discounted Magni risk (CDMR):

$$CDMR\left(b_{t}\right)=\sum_{i=0}^{h-1}\gamma^{i}\times MR\left(b_{t+i}\right)$$

$$\bar{\underline{\begin{array}{l} \underline{Algorithm\;1\text{:}\;\text{Psuedocode}\;\text{of}\;\text{Control}\;\text{Algorithm}.\;\;\;\;\;}\\ \;Input:\;\text{forecaster}\;f\text{,}\;\text{action}\;\text{distribution}\;𝓐\text{,}\\ \;\;\text{\#}\;\text{of}\;\text{candidate}\;\text{actions}\;N\text{,}\;\text{risk}\;\text{function}\;MR\\ \;Variables:\\ \;\;t\text{:}\;\text{Time}\;\text{point}\\ \;\;CHO_{t-1}\text{:}\;\text{Carbohydrate}\;\text{given}\;\text{at}\;\text{time}\;\text{point}\;t-1\\ \;\;a_{t}^{*}\text{:}\;\text{Best}\;\text{action}\;\text{at}\;\text{time}\;\text{point}\;t\\ \;while\;Running\;do\\ \;\;a_{t}^{*}=0\\ \;\;if\;CHO_{t-1}>0\;then\\ \;\;\;for\;i\in \left[1,\;\ldots ,\;N\right]\;do\\ \;\;\;\;a_{i}∼𝓐\;\;\;\;\;\;⊳\text{Select}\;\text{random}\;\text{action}\\ \;\;\;\;{𝓣}_{i}=f\left(a_{i}\right)\;\;\;\;\;⊳\text{Generate}\;\text{BG}\;\text{forecast}\\ \;\;\;end\;for\\ \;\;\;a_{t}^{*}=\arg\min_{a_{i}}MR\left({𝓣}_{i}\right)\;⊳\text{Action}\;\text{with}\;\text{lowest}\;\text{risk}\\ \;\;end\;if\\ \;\;\text{Execute}\;\text{action}\;a_{t}^{*}\\ \;end\;while \end{array}}}$$

where the discount factor γ=0.99. This process is repeated for every time step. A 4-hour forecast is needed as shorter predictions fail to fully capture the delayed effects of insulin and can lead to over-administration of insulin.

### D. Evaluation

#### 1) Evaluation Under Behavior Policy:

The standard method by which glucose forecasters are currently evaluated is to measure the forecast error under the behavior policy. Error was measured in terms of RMSE and mean absolute error (MAE) on 15 days of held-out test data per individual. RMSE and MAE are measured at multiple prediction horizons within the forecast. For a prediction horizon h, RMSE and MAE are calculated as:

$$RMSE\left(f,\;h,\;D\right)=\sqrt{\frac{1}{\left|D\right|}\sum_{t\in D}{\left(f_{t+h}-y_{t+h}\right)}^{2}}$$

$$MAE\left(f,\;h,\;D\right)=\frac{1}{\text{|}D\text{|}}\sum_{t\in D}\left|f_{t+h}-y_{t+h}\right|$$

where f is the forecaster, D is the test data, $f_{t+h}$ is the $h^{\text{th}}$ point in the forecast made by f at time t, and $y_{t+h}$ is the true BG value at time t+h. We measured the metrics at prediction horizons ranging from 30 minutes to 4 hours and calculated the mean, 95% confidence interval, and standard error across 1000 bootstraps. We chose these error metrics due to their widespread use in BG forecast evaluation [9], [10].

#### 2) Evaluation Under MBC Setting:

We test our hypothesis that forecasters trained and evaluated under the behavior policy may perform poorly in MBC settings by evaluating both the forecast error under counterfactual actions and downstream control performance. 100 days of test data for each individual were generated using each forecaster and the MBC control algorithm (20 rollouts of 5 consecutive days). Forecast error was measured in terms of RMSE and MAE over all candidate trajectories tested during the planning step. To ensure a fair comparison, we measured the forecast error on a shared evaluation dataset that contained 5000 candidate trajectories from each forecaster.

Downstream control performance was measured in terms of % time-in-range (70 − 180 mg/dL; TIR), % time-below-range (< 70 mg/dL; TBR), and % time-above-range (> 180 mg/dL; TAR) daily, which are standard in assessing the quality of BG management [37], [38], along with daily average Magni risk. We reported the median and interquartile range across individuals.

#### 3) Visualizing Isolated Effects:

We also visualized the BG forecasts when only insulin or carbohydrates (not both) were present in the input window. The predictions were obtained through separate simulations where the patient received either carbohydrates or insulin in isolation. Isolated meal and bolus sizes were set as the average value of each variable in the standard dataset generated under the behavior policy. This tests the impulse response of the forecaster to insulin and carbohydrates and can serve as a qualitative proxy in determining whether the causal effects of insulin and carbohydrates were captured in the model. To understand the effect of each variable on BG, we measured the deviation of the BG predictions from the last recorded BG value and compared them to the true BG trajectories obtained from the simulator.

## III. Results

### A. Evaluation Under Behavior Policy

**Fig. 1.**
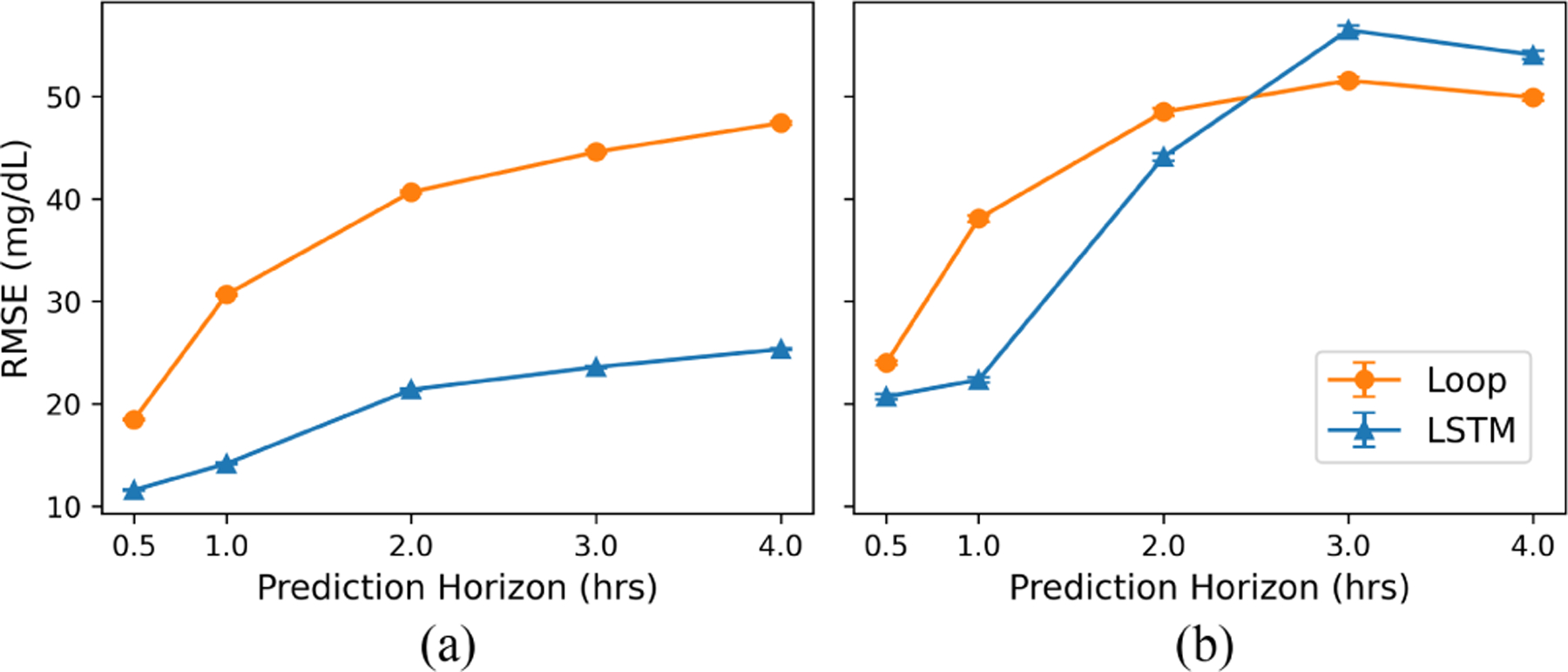
Average forecast error (in RMSE) under (a) behavior policy and (b) MBC setting across 1000 bootstraps. Error bars indicate the 95% confidence interval. LSTM had better forecast error than Loop under the behavior policy. However, this trend was reversed under the MBC setting with LSTM exhibiting worse forecast performance than Loop, especially towards the end of the forecast.

Under the behavior policy, LSTM consistently achieved significantly better (lower) forecast error than Loop across all prediction horizons (Fig. 1(a)). At 30 minutes, LSTM had an RMSE of 11.57 ± 0.05, which is comparable to state-of-the-art ML forecasters [9]. By contrast, Loop had an RMSE of 18.46 ± 0.07. This gap in performance increases with the length of the prediction horizon, a trend that is also reflected in MAE measurements (Appendix C).

### B. Evaluation Under MBC Setting

**Fig. 2.**
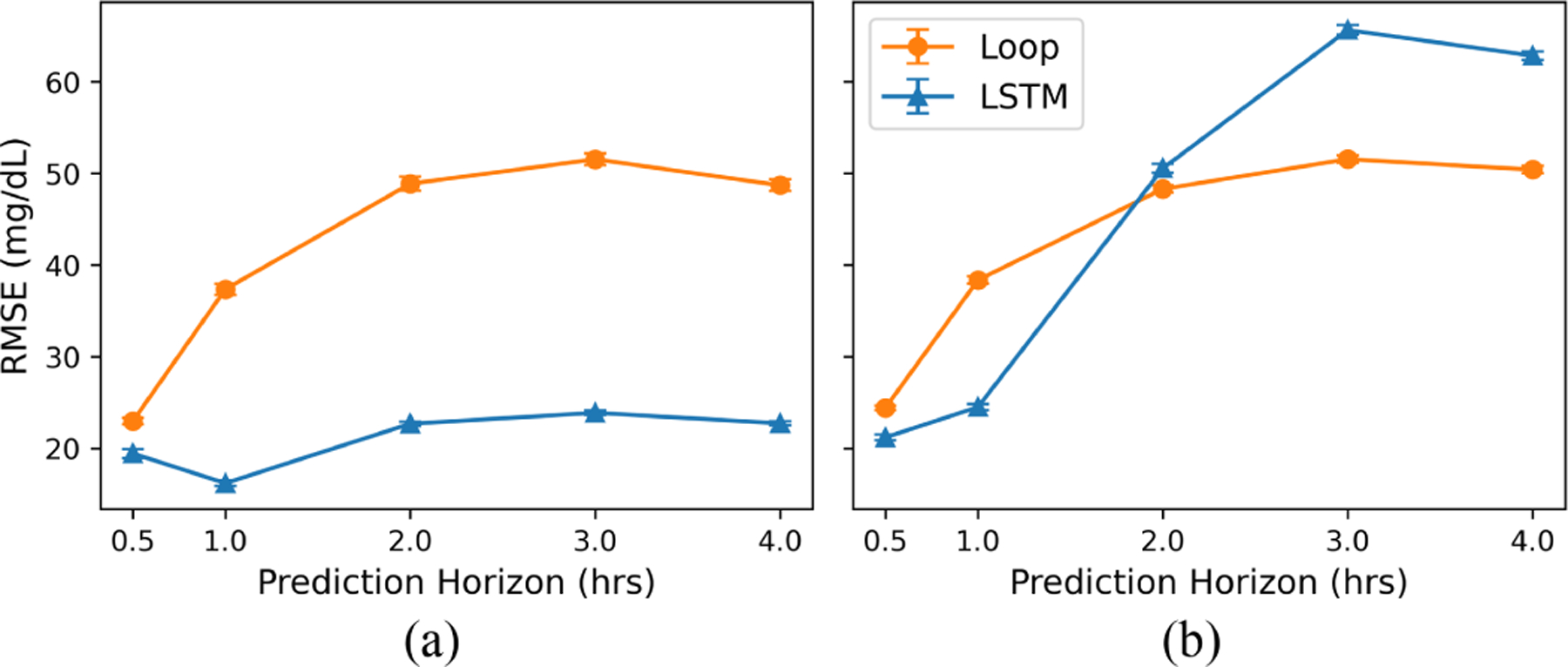
Average forecast error (in RMSE) under MBC setting for datasets (a) within the training distribution and (b) outside the training distribution. Error bars indicate the 95% confidence interval. LSTM performs well for carbohydrate-insulin pairs seen during training but exhibits worse performance for pairs outside the training distribution.

However, in sharp contrast, LSTM’s forecast error significantly increased in the MBC setting and displayed poorer accuracy compared to Loop for longer prediction horizons (Fig. 1(b)). While LSTM had a lower forecast error than Loop for shorter prediction horizons (RMSE at 30 minutes: 20.69 vs. 23.98), LSTM’s error increased as the prediction horizon increased. Comparison of predictions at the 4-hour time point shows that the forecast ranking of the two forecasters is flipped, with Loop achieving a significantly lower RMSE of 49.91 ± 0.34 compared to 54.06 ± 0.39 for LSTM. Since the control algorithm relies on longer prediction horizons due to the delayed nature of insulin and carbohydrate effects, the accuracy of the forecasters for these longer prediction horizons can have a significant impact on the quality of the control decisions. We further divided the test set into predictions generated from carbohydrate-insulin pairs, in which the pairs were eisther within (Fig. 2(a)) or outside (Fig. 2(b)) the behavior policy. We see that while LSTM can accurately predict the effects of carbohydrate-insulin pairs it has encountered during training, it fails to generalize to pairs outside the training distribution. For example, at the 4-hour time point, RMSE was 22.73 ± 0.22 for trajectories within the training distribution but 62.83 ± 0.46 for those outside the training distribution. By contrast, Loop’s forecast error, while generally on the higher end, is consistent across the entire dataset (RMSE 48.69 ± 0.62 vs. 50.40 ± 0.40 at 4 hours).

Worse forecast performance under the MBC setting also translated to lower downstream control performance (Table I). Loop achieved 86.20% TIR (IQR: 78.28 − 91.21) corresponding to an average Magni risk of 4.95 across the patient cohort. By comparison, LSTM was significantly lower, achieving 77.14% TIR (IQR: 66.57 − 84.03, *p* < 0.05), and an average Magni risk of 7.36. It also led to higher incidents of hypoglycemia (TBR) which is widely considered to be more dangerous than hyperglycemia (TAR). These results demonstrate that better accuracy in current evaluation approaches that rely only on test data collected under a behavior policy does not necessarily translate into better downstream control.

### C. Visualizing Isolated Effects

**Fig. 3.**
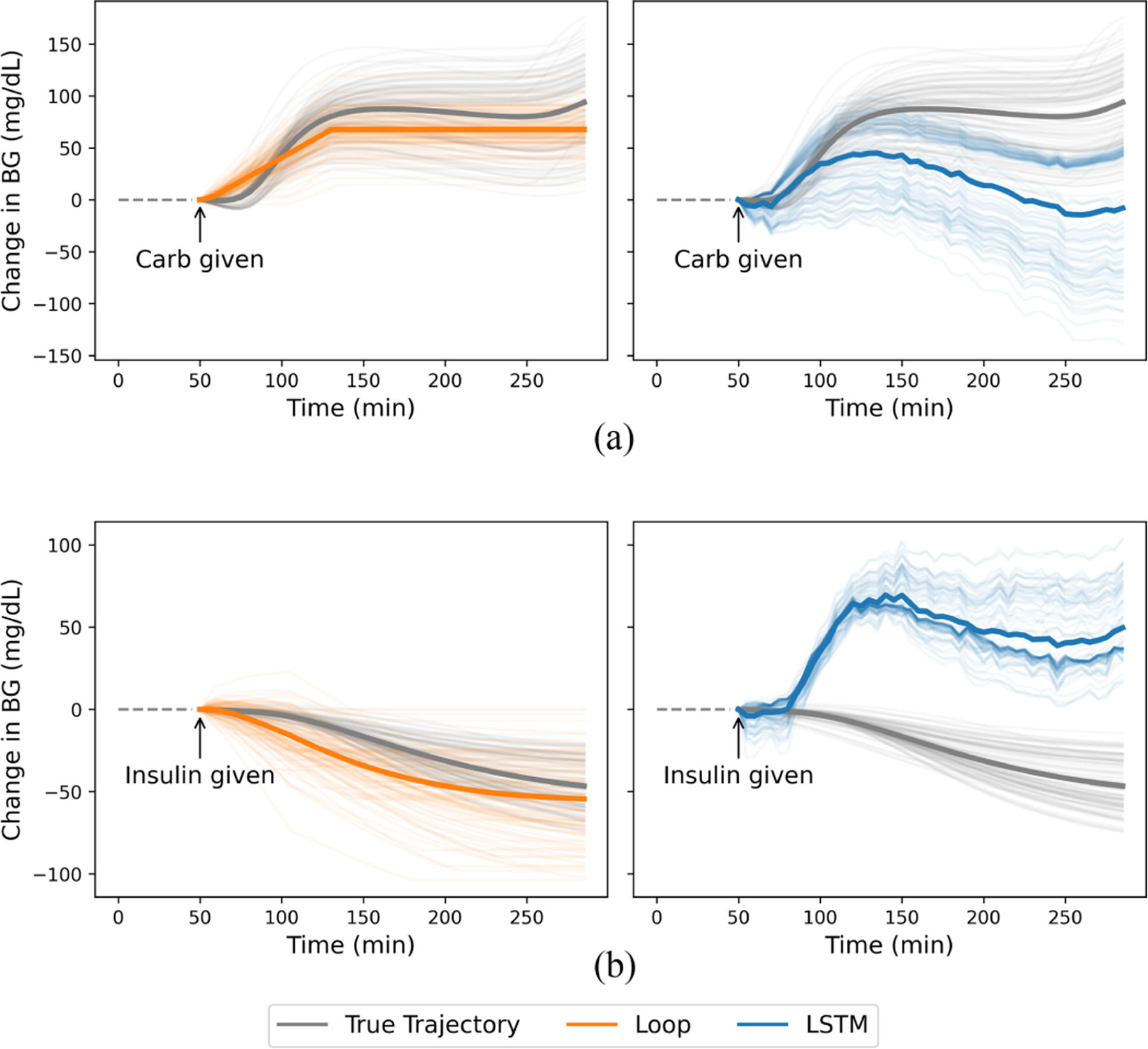
100 example predictions made by the forecasters immediately after (a) carbohydrate or (b) insulin intake. Mean trajectories are indicated in bold. Grey lines indicate the true trajectory generated using the simulator. Loop’s (left panel) response to carbohydrates and insulin aligns closely with the true trajectories. However, LSTM (right panel) underestimates the increase in BG from carbohydrates and predicts that BG will increase rather than decrease from insulin.

Fig. 3 shows the impulse response of Loop and LSTM to carbohydrates (Fig. 3(a)) and insulin (Fig 3(b)). Loop generated predictions that aligned with clinical understanding, where insulin leads to a decrease and carbohydrates lead to an increase in BG. However, LSTM tended to generate predictions that underestimated or even inverted the effect of each variable. This demonstrates that LSTM failed to capture the causal effects of each variable.

## IV. Discussion

Our analyses demonstrate that the current approach of evaluating BG forecasters is not sufficient when selecting models for closed-loop control. While ML models such as LSTM have achieved lower forecast error on datasets that are within the training distribution, this is not necessarily representative of the data the model might encounter when paired with a model-based controller. Forecasters can encounter out-of-distribution data during planning, specifically carbohydrate-bolus pairs that do not occur under the behavior policy. Fig. 2(b) shows that LSTM generates highly inaccurate predictions for these counterfactual actions, leading to suboptimal action selection. Despite current evaluation practices favoring LSTM over Loop, our results demonstrate that this can lead to catastrophic control decisions.

We hypothesize that LSTM fails to generalize because of the strong correlation between insulin and carbohydrates in the training data. These variables are highly correlated in terms of magnitude, as they are typically administered together in a basal-bolus dosing regimen where the bolus size is dependent on the amount of carbohydrates. This prevents the model from learning the individual effects of each variable, resulting in a conflation of their effects as demonstrated by the trajectories in Fig. 3. This conflation makes it challenging for LSTM to generate accurate predictions for all the combinations of carbohydrates and insulin that are considered during planning. By contrast, Loop correctly predicts the individual effects, because both carbohydrates and insulin are explicitly modeled based on domain knowledge. While we compare LSTM and Loop as a simple example to demonstrate the failings of current evaluation methods, it is important to note that we are not making any claims regarding the general effectiveness of ML-based vs. rule-based forecasters for model-based control. Trained to accurately predict the individual effects of carbohydrates and insulin, we expect ML-based approaches with appropriate model selection to ultimately outperform rule-based forecasters in control settings.

While we demonstrated our findings on simulated data, this problem exists in real data as well. Benchmark clinical datasets, such as the Ohio T1DM dataset, use data generated from users using standard insulin pump therapy and exhibit the same high correlation between insulin and carbohydrates [12] (Supplement Fig. 1). Thus we expect ML models trained on real data to also suffer from the same shortcomings as our LSTM forecaster. Analyzing the predictions of the forecasters when only carbohydrates or insulin is present in the input as we have done in Fig. 3 could aid in identifying these shortcomings. Specifically, models must be evaluated on their ability to predict accurate trajectories for counterfactual actions. We encourage researchers to explore additional methods to perform a more rigorous evaluation of BG forecasters.

Our study is not without limitations. First, due to experimental constraints, the MBC portion of our analysis was limited to 10 virtual adults. Future studies should verify whether these findings remain consistent in real individuals and across broader age groups. Second, while we used a single control algorithm and limited it to a bolus advisor, successful commercialization of hybrid closed-loop systems such as Tandem IQ or Medtronic Automode indicates that other control algorithms that target glucose control optimization are available. While we hypothesize that our findings hold for any control algorithm that utilizes a trajectory optimization method (i.e., select the optimal action from a set of actions by simulating the trajectory of each action) with long prediction horizons, a future study is necessary to test this hypothesis. In addition, we note that our findings are limited to forecasters trained on data collected in open-loop settings (i.e., basal-bolus insulin therapy) where a high correlation exists between carbohydrate and insulin events. However, this issue may not be as pronounced in forecasters trained on closed-loop data collected under a different insulin dosing policy with less correlation between carbohydrates and insulin. Finally, due to the safety-critical nature of the domain, our control experiments were limited to simulated environments. Further research is required to validate the existence of similar issues in control settings and its downstream impact for forecasters trained and evaluated on real-word data.

While there has been a recent uptick in interest in applying AI/ML techniques to BG forecasting and management [10], [22], much of the AI/ML community has focused on a limited set of evaluation metrics that prioritizes minimizing RMSE for short-term prediction horizons on in-distribution samples only. We show that by neglecting to evaluate on out-of-distribution data that forecasters may encounter during planning, ML researchers risk over-optimizing their forecasting models for a setting that does not necessarily lead to better BG management. Instead of optimizing for a single evaluation metric, we encourage researchers to adopt a holistic evaluation approach that moves beyond overall RMSE when selecting models for model-based control.

Our work augments previous efforts to raise awareness of the issue of collinearity in carbohydrates and insulin in commonly used open-loop datasets, and the negative impact it can have on machine learning forecasters. We do so by demonstrating the limitations of current evaluation approaches and highlighting the downstream impact on model selection. Future work should explore additional evaluation metrics and training strategies to develop and select predictive models for closed-loop control.

## V. Conclusion

The prevailing evaluation standard for BG forecasters is to measure the models’ forecast error on benchmark datasets generated under the behavior policy. Our study demonstrates that such evaluation approaches are insufficient, particularly in recognizing cases where the forecast model conflates the effects of carbohydrates and insulin. Our work shows that when developing forecast models for use in hybrid closed-loop systems for BG management, researchers must evaluate them using data representative of real-world planning scenarios. Furthermore, we encourage researchers to evaluate these forecasters for their ability to accurately predict the independent effects of carbohydrates intake and insulin dosing. Such careful evaluation is paramount prior to deploying hybrid closed-loop systems but can also inform research directions within the community of machine learning practitioners working on BG forecasting algorithms.


$$\bar{\underline{\begin{array}{l} \underline{Algorithm\;2\text{:}\;\text{Meal}\;\text{Schedule}\;\text{Generation}.\;\;\;\;\;\;\;}\\ \;Input:\;\text{body}\;\text{weight}\;w\text{,}\;\text{age}\;a\text{,}\;\text{height}\;h\text{,}\;\text{\#}\;\text{of}\;\text{days}\;n\\ \;\text{BMR}=66.5+\left(13.75\times w\right)+\left(5.003\times h\right)-\left(6.755\times a\right)\\ \;\text{ExpectedCarbs}=\left(\text{BMR}\times 0.45\right)\text{/}4\\ \;\text{MealOcc}=\left[0.95,\;0.95,\;0.95\right]\\ \;\text{TimeLowerBounds}\;=\left[5,\;10,\;16\right]\\ \;\text{TimeUpperBounds}\;=\left[9,\;14,\;20\right]\\ \;\text{TimeMean}\;=\left[7,\;12,\;18\right]\\ \;\text{TimeStd}\;=\left[1,\;1,\;1\right]\\ \;\text{AmtMean}\;=\left[0.333,\;0.333,\;0.334\right]*\text{ExpectedCarbs}*1.2\\ \;\text{AmtStd}\;=\text{AmtMean}*0.15\\ \;\text{Schedule}\;=[\;]\\ \;for\;i\in \left[1,\ldots ,n\right]\;do\;\\ \;\;for\;j\in \left[1,\;2,\;3\right]do\\ \;\;\;m∼Binomial\left(\text{MealOcc}\left[j\right]\right)\\ \;\;\;lb=\text{TimeLowerBounds}\left[j\right]\\ \;\;\;ub=\text{TimeUpperBounds}\left[j\right]\\ \;\;\;\mu_{t}=\text{TimeMean}\left[j\right]\\ \;\;\;\sigma_{t}=\text{TimeStd}\left[j\right]\\ \;\;\;\mu_{a}=\text{AmtMean}\left[j\right]\\ \;\;\;\sigma_{a}=\text{AmtStd}\left[j\right]\\ \;\;\;if\;m\;\text{is}\;\text{True}\;\text{then}\\ \;\;\;\;\text{Time}∼Round\left({𝓝}_{trunc}\left(\mu_{t},\;\sigma_{t},\;lb,\;ub\right)\right)\\ \;\;\;\;\text{carbAmount}∼Round\left(\max\left(0,\;𝓝\left(\mu_{a},\;\sigma_{a}\right)\right)\right)\\ \;\;\;\;M=\left(\text{Time},\;\text{carbAmount}\right)\\ \;\;\;\;\text{Schedule}.\text{append}\left(M\right)\\ \;\;\;end\;if\\ \;\;end\;for\\ \;end\;for \end{array}}}$$

## Supplementary Material

supp1-3424665

## Figures and Tables

**Fig. 4.**
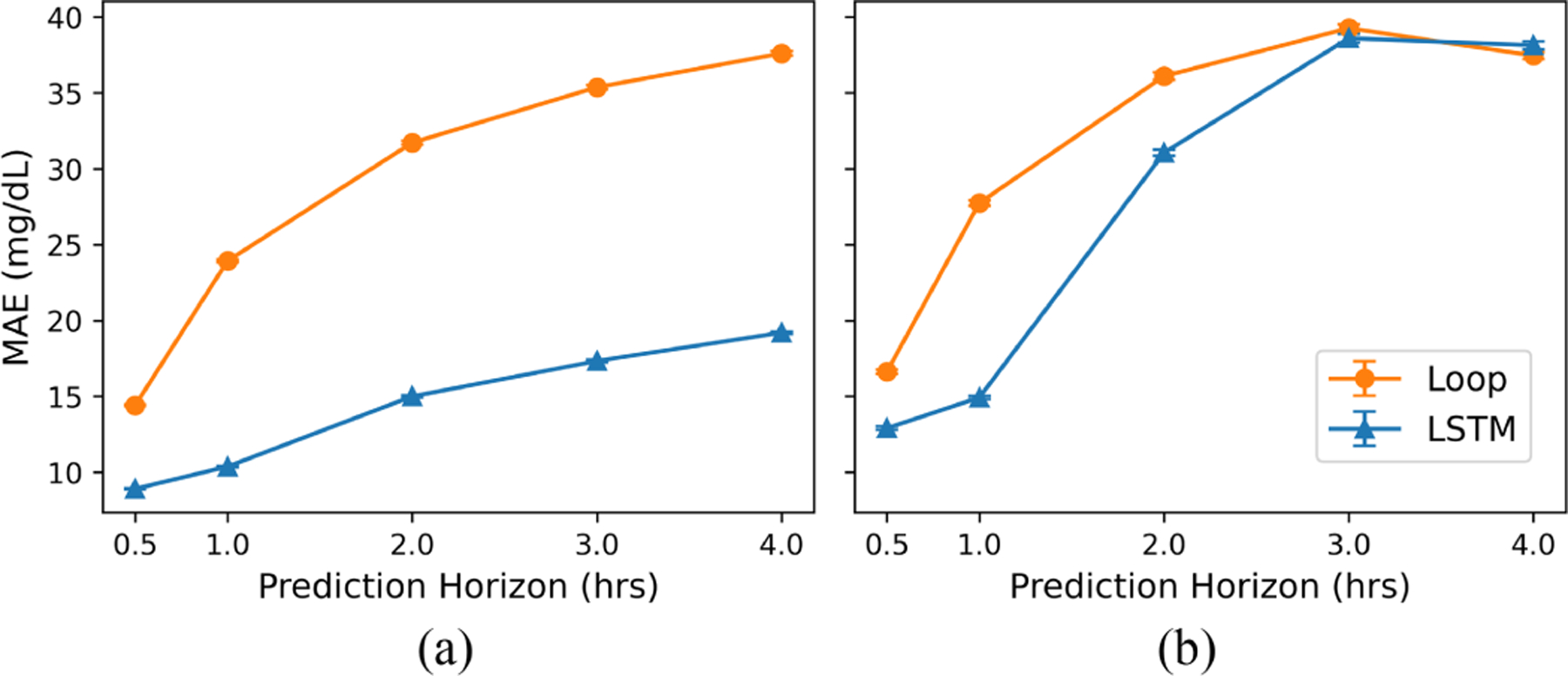
Average forecast error (in MAE) under (a) behavior policy and (b) MBC setting across 1000 bootstraps. Error bars indicate the 95% confidence interval.

**Fig. 5.**
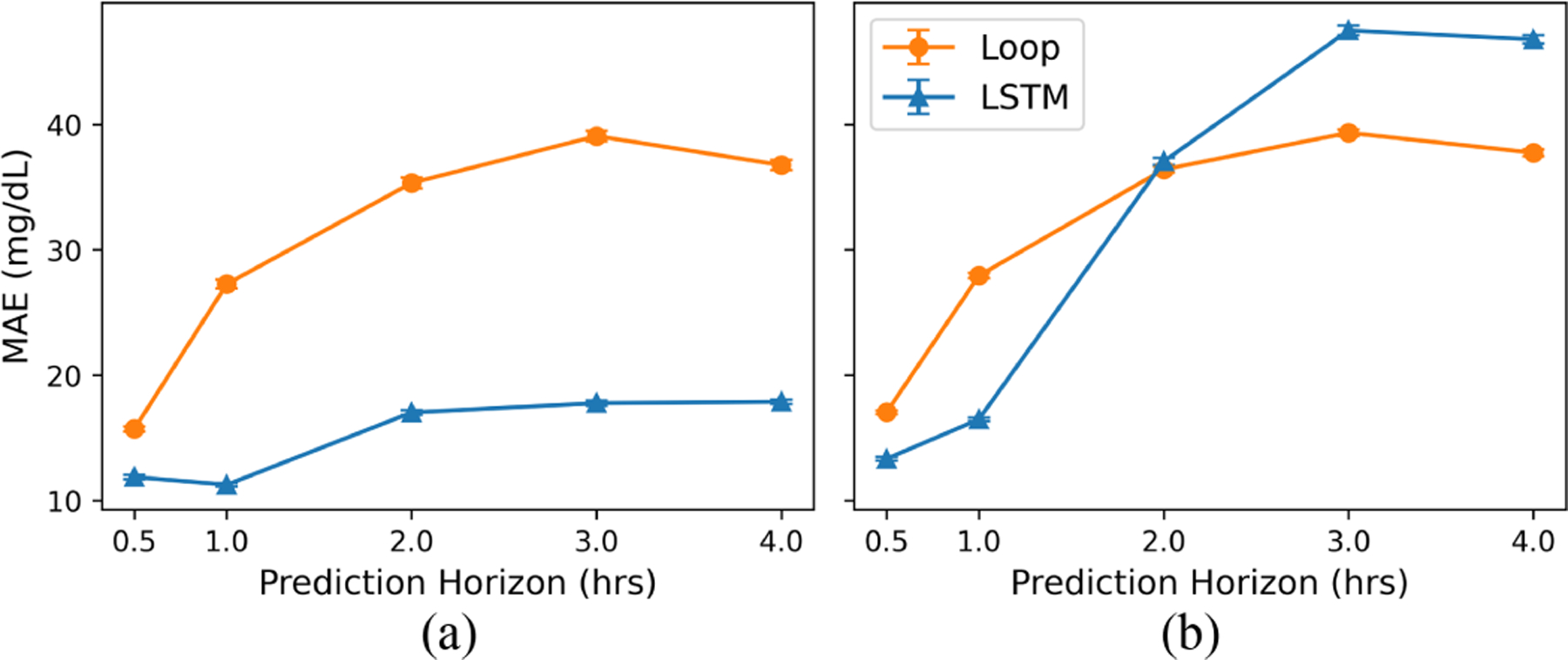
Average forecast error (in RMSE) under MBC setting for datasets (a) within the training distribution and (b) outside the training distribution. Error bars indicate the 95% confidence interval.

**TABLE I:** Comparison of Forecasters’ Median Control Performance in MBC Setting Across All Patients

Forecaster	% TIR ↑	% TAR ↓	% TBR ↓	MR ↓
Loop	**86.20** (78.28, 91.21)	**10.07** (6.23, 18.60)	**2.56** (0.00, 5.90)	**4.95** (3.33, 6.78)
LSTM	77.14 (66.57, 84.03)	17.42 (10.27, 27.14)	4.34 (0.00, 10.09)	7.36 (5.26, 9.84)

TIR: time in range; TAR: time above range; TBR: time below range; MR: Magni risk Values in parantheses indicate interquartile range. Loop obtains better BG control across all metrics compared to LSTM.

**TABLE II:** Hyperparameter Values Considered for LSTM

Hyperparameter	Values
Length of forecast	6, 12, 48
Length of input	24, 36, 48
IOB, COB estimates in input	True, False
# of hidden states	16, 32, 64, 128, 256
# of layers	1, 2
Batch size	256, 512, 1024
Learning rate	10^−2^, 10^−3^, 10^−4^, 10^−5^

IOB: insulin-on-board; COB: carbohydrates-on-board;

## Appendix

### A. Meal Schedule

We used Algorithm 2 to generate the meal schedule. Basal metabolic rate is calculated using the Harris-Benedict equation [39] and further used to estimate the expected daily carbohydrate intake assuming a diet where 45% of calories come from carbohydrates [36]. The daily carbohydrate intake is divided between 3 meals each day: breakfast, lunch, and dinner.

### B. LSTM Model Training

The data was preprocessed by clipping blood glucose values to be between [40, 400] and multiplying it by 0.01. Carbohydrate values were divided by 20 and total insulin amounts were multiplied by 10. These constants were chosen as they approximately normalized the values of carbohydrates and insulin to be within the range [0, 1]. Table II lists all hyperparameters tested. The model with the best validation RMSE was chosen for each patient. All models were implemented in PyTorch and trained using an Adam optimizer, with an early stopping of 20 epochs’ patience. We explored models that generate 30-minute or 60-minute forecasts, which were more commonly explored in previous literature, but found that the 4-hour forecast model led to the best overall performance.

### C. MAE Results

We report the results for forecast error measured in terms of MAE in Figs. 4 and 5. The overall trend aligns with those observed from RMSE. Results for additional metrics such as mean absolute relative difference (MARD), gluco-specific RMSE (gRMSE) [40], and Clarke error grid [41] can be found in the supplement (Supplement Figs. 1 and 2, Table 1).
