# Supplementary material for: Shortcomings in the Evaluation of Blood Glucose Forecasting
Source: IEEE Trans Biomed Eng. Author manuscript; Available in PMC 2025 Mar 4. (PMC11724010; doi:10.1109/TBME.2024.3424665)
Supplement: supp1-3424665 [file NIHMS2037597-supplement-supp1-3424665.pdf]

Supplementary Material

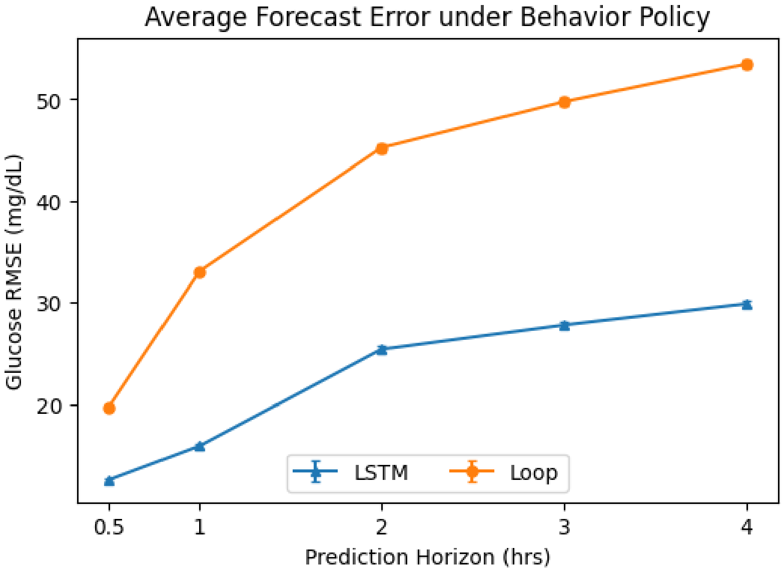

Supplement Figure 1. Average and 95% confidence interval of the forecast error in mean absolute relative difference (MARD) measured across 1000 bootstraps of the test data for LSTM and Loop forecasters under the behavior setting.

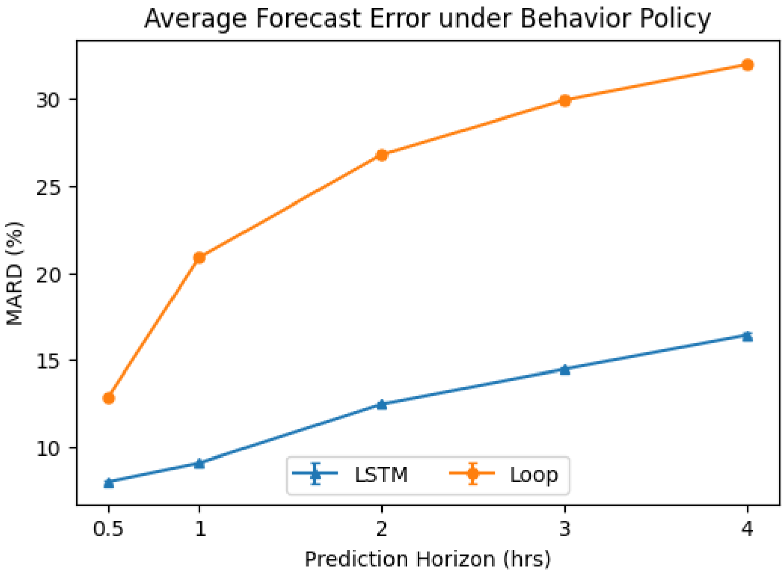

Supplement Figure 2. Average and 95% confidence interval of the forecast error in gluco-specific RMSE (gRMSE) measured across 1000 bootstraps of the test data for LSTM and Loop forecasters under the behavior setting.

Supplement Table 1. Clarke error grid analysis for LSTM and Loop forecasters for predictions made under the behavior policy. Evaluated at prediction horizons ranging from 30 minutes to 4 hours.

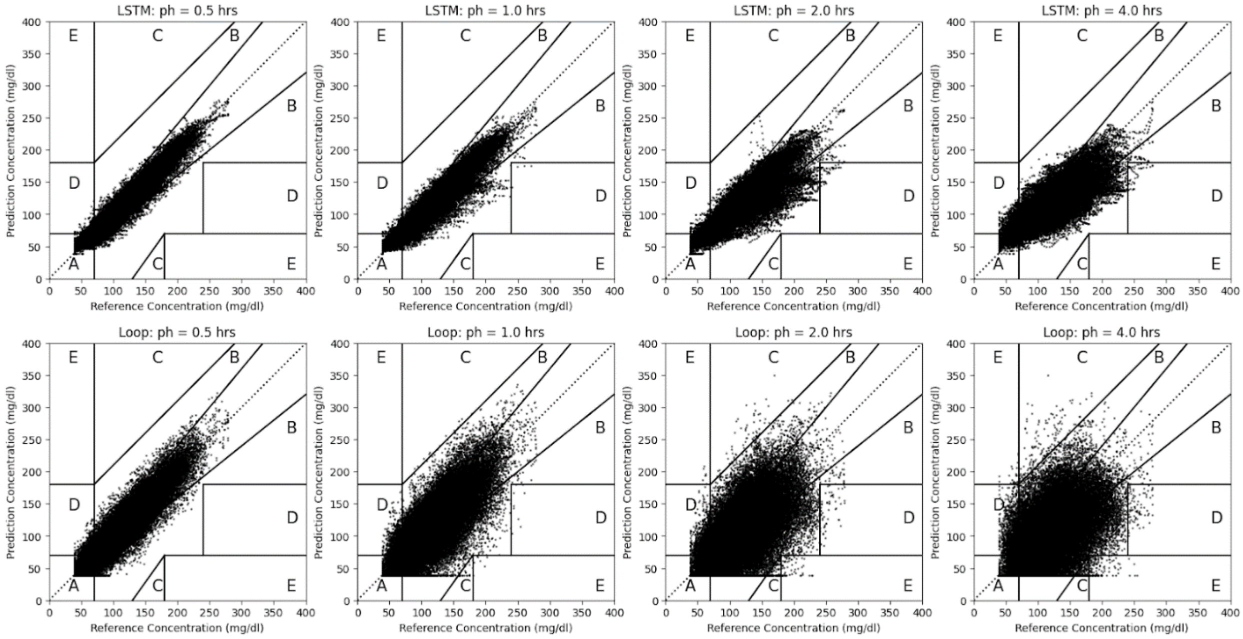

Prediction Horizon	Forecaster	Zone A (%)	Zone B (%)	Zone C (%)	Zone D (%)	Zone E (%)
30 min	LSTM	94.8	4.4	0.0	0.9	0.0
	Loop	81.5	16.6	0.0	1.9	0.0
1 hour	LSTM	91.9	7.1	0.0	1.0	0.0
	Loop	59.3	37.5	0.1	3.1	0.0
2 hours	LSTM	81.1	16.4	0.0	2.5	0.0
	Loop	47.7	48.4	0.4	3.3	0.2
4 hours	LSTM	69.7	25.9	0.0	4.5	0.0
	Loop	40.2	54.6	0.9	4.0	0.4

Supplement Figure 3. Distribution of insulin and carbohydrates in OhioT1DM dataset. The color represents the relative frequency of the carbohydrate amount and corresponding bolus doses for the combined Ohio T1DM dataset at different BG levels. Numbers on the left hand corner of each panel represent the Pearson correlation of the carbohydrate and bolus amounts.

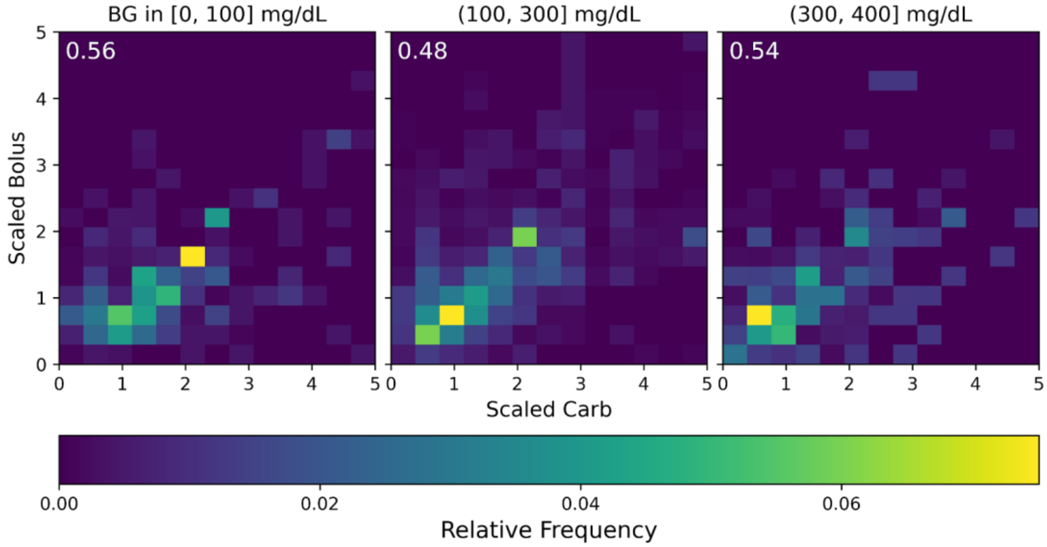

Supplement Table 2. Median control performance of Oracle forecaster across all patients. Values in parentheses indicate interquartile range.

Forecaster	%TIR	%TAR	%TBR	MR
Oracle	92.83 (87.80, 96.44)	4.29 (1.04, 10.26)	0.69 (0.00, 4.73)	3.35 (1.95, 5.29)
